# Discovery of pre-therapy 2-deoxy-2-^18^F-fluoro-D-glucose positron emission tomography-based radiomics classifiers of survival outcome in non-small-cell lung cancer patients

**DOI:** 10.1007/s00259-018-4139-4

**Published:** 2018-09-01

**Authors:** Mubarik A. Arshad, Andrew Thornton, Haonan Lu, Henry Tam, Kathryn Wallitt, Nicola Rodgers, Andrew Scarsbrook, Garry McDermott, Gary J. Cook, David Landau, Sue Chua, Richard O’Connor, Jeanette Dickson, Danielle A. Power, Tara D. Barwick, Andrea Rockall, Eric O. Aboagye

**Affiliations:** 1 Imperial College London Cancer Imaging Centre, Department of Surgery & Cancer, Hammersmith Hospital, Du Cane Road, London, W12 0NN UK; 2 Imperial College Healthcare NHS Trust, Departments of Clinical Oncology, Radiology and Nuclear Medicine, Hammersmith Hospital, Du Cane Road, London, W12 0HS UK; 3 Charing Cross Hospital, Fulham Palace Road, London, W6 8RF UK; 4 Department of Nuclear Medicine, Level 1, Bexley Wing, St James’s University Hospital, Beckett Street, Leeds, LS9 7TF UK; 5 Leeds Institute of Cancer and Pathology, School of Medicine, University of Leeds, Leeds, UK; 6 Department of Cancer Imaging, School of Biomedical Engineering and Imaging Sciences, King’s College London, St. Thomas’ Hospital, Westminster Bridge Rd, London, SE1 7EH UK; 7 Department of Nuclear Medicine, The Royal Marsden Hospital, Downs Rd, Sutton, London, SM2 5PT UK; 8 Department of Nuclear Medicine, Queen’s Medical Centre, Nottingham University Hospital, Derby Rd, Nottingham, NG7 2UH UK; 9 Department of Clinical Oncology, Mount Vernon Hospital, Rickmansworth Road, Northwood, HA6 2RN UK

**Keywords:** Radiomics, NSCLC, Survival, PET, Risk stratification

## Abstract

**Purpose:**

The aim of this multi-center study was to discover and validate radiomics classifiers as image-derived biomarkers for risk stratification of non-small-cell lung cancer (NSCLC).

**Patients and methods:**

Pre-therapy PET scans from a total of 358 Stage I–III NSCLC patients scheduled for radiotherapy/chemo-radiotherapy acquired between October 2008 and December 2013 were included in this seven-institution study. A semi-automatic threshold method was used to segment the primary tumors. Radiomics predictive classifiers were derived from a training set of 133 scans using TexLAB *v2*. Least absolute shrinkage and selection operator (LASSO) regression analysis was used for data dimension reduction and radiomics feature vector (FV) discovery. Multivariable analysis was performed to establish the relationship between FV, stage and overall survival (OS). Performance of the optimal FV was tested in an independent validation set of 204 patients, and a further independent set of 21 (TESTI) patients.

**Results:**

Of 358 patients, 249 died within the follow-up period [median 22 (range 0–85) months]. From each primary tumor, 665 three-dimensional radiomics features from each of seven gray levels were extracted. The most predictive feature vector discovered (FVX) was independent of known prognostic factors, such as stage and tumor volume, and of interest to multi-center studies, invariant to the type of PET/CT manufacturer. Using the median cut-off, FVX predicted a 14-month survival difference in the validation cohort (*N* = 204, *p* = 0.00465; HR = 1.61, 95% CI 1.16–2.24). In the TESTI cohort, a smaller cohort that presented with unusually poor survival of stage I cancers, FVX correctly indicated a lack of survival difference (*N* = 21, *p* = 0.501). In contrast to the radiomics classifier, clinically routine PET variables including SUV_max_, SUV_mean_ and SUV_peak_ lacked any prognostic information.

**Conclusion:**

PET-based radiomics classifiers derived from routine pre-treatment imaging possess intrinsic prognostic information for risk stratification of NSCLC patients to radiotherapy/chemo-radiotherapy.

**Electronic supplementary material:**

The online version of this article (10.1007/s00259-018-4139-4) contains supplementary material, which is available to authorized users.

## Introduction

Lung malignancy is a leading cause of cancer-related death, with a predicted 5-year survival rate of 8–13% [1]. Worldwide, approximately 1.8 million new cases were diagnosed in 2012. Distinct from histology, stage, and performance status, the ability to provide prognosis on the basis of tumor biology is often lacking in current clinical practice. More recently, DNA sequencing from several tumor regions has been undertaken to highlight spatio-temporal mutational heterogeneity [2, 3]. Currently, imaging identifies the sites of disease and response to treatment by assessing the change in size but other than TNM staging provides limited prognostic information. In addition, outcomes of patients within each TNM staging group can vary widely highlighting the need for more accurate prognostic markers. Potential interventional methods to assess genetic heterogeneity will probably employ multi-core invasive biopsy, which limits its safe use for routine prognosis determination. The micro- and macro-structure of tumors, however, also harbor heterogeneous phenotypes due to factors such as hypoxia, necrosis, directional/non-directional tumor cell growth, vascular density, and immune infiltration. It is hypothesized that the asymmetric local, regional, and global density and architectural distortions of tumor phenotypes could have prognostic value, and this has resulted in a new 'omics' — radiomics [4–6] — whereby quantitative features describing tumor phenotypes are extracted in high-throughput from routine radiologic images and further processed by machine learning methods for prognostication; such high-dimensional output of tumor phenotypic heterogeneity is thought to have important prognostic value, with drug resistance and potential for development of metastatic spread implied.

2-deoxy-2-^18^Fluorine-fluoro-D-glucose positron emission tomography-computed tomography (FDG-PET/CT) is routinely used for staging lung cancer prior to consideration of radical treatment such as surgery or chemo-radiotherapy including the use of stereotactic body radiotherapy. Indeed, radiomics classifiers based on the CT component have been investigated for predicting lung cancer histology and shown to have moderate prediction accuracy [7]. Beyond the use of FDG-PET/CT for staging, we investigated in the present study whether pre-therapy radiomics features derived from routine FDG-PET/CT examinations of non-small-cell lung cancer (NSCLC) patients who were subsequently treated with radiotherapy/chemo-radiotherapy across multiple hospitals might harbor useful prognostic information.

## Patients and methods

### Patients and procedures

The inclusion criteria were consecutive patients with non-small-cell lung cancer (NSCLC), or entire available cohort for The Cancer Imaging Archive (TCIA) (http://cancerimagingarchive.net/, last accessed June 2015), with a target lesions ≥ 5 ml who had a pre-therapy FDG-PET/CT scan available and underwent radical radiotherapy with or without chemotherapy between October 2008 and December 2013. The minimum lesion volume of interest (VOI) of 5 ml was selected, in accordance with work carried out by Soussan et al. [8]. Exclusion criteria were patients undergoing surgery or palliative treatment. Institutional ethical approval for retrospective analysis was obtained, and informed consent was waived.

**Fig. 1:**
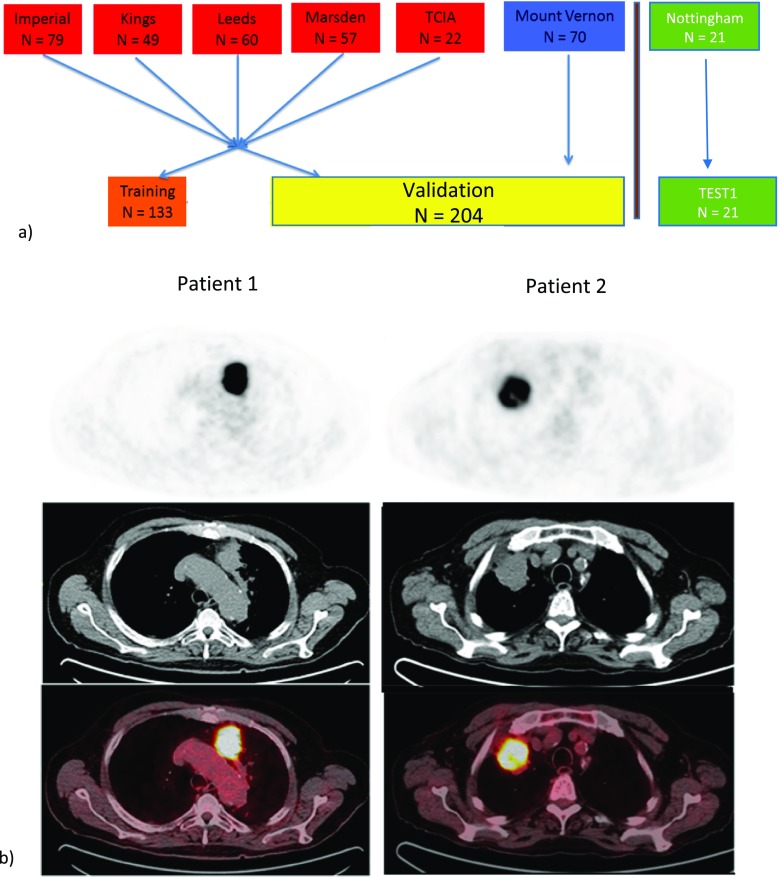
Overview of centers and PET images.**a** Overview of the centers contributing to the study and how the data were randomly divided into training, validation or independent test set. TCIA, The Cancer Imaging Archive. **b** Typical images from the PET/CT scans of two patients including PET, CT, and fusion images. Patient 1 (age 74, squamous cell carcinoma, stage IIA, tumour volume 22.6, overall survival 8 months) with the lower stage and smaller volume primary lesion had a worse survival outcome than patient 2 (age 77, squamous cell carcinoma, stage IIIA, tumour volume 26.5, overall survival 33 months) with the higher tumour stage

The following hospitals took part in the trial (Fig. 1): Imperial College Healthcare NHS Trust, London, St James’s University Hospital, Leeds, Guy’s and St. Thomas’ Hospitals, London, The Royal Marsden Hospital, Sutton, Nottingham University Hospital, Nottingham, and Mount Vernon Hospital, Northwood; a dataset was also obtained from TCIA. This work was carried out sequentially with training and validation followed by TESTI. Data from the four hospitals and The Cancer Imaging Archive (Imperial, Kings, Leeds, and Royal Marsden, and TCIA) patients were collated and randomly split into two (by computer) as training set (*n* = 133) and validation set (*n* = 134). A power calculation based on the training set (HR = 1.78, median survival: 2.92 years, censoring rate: 0.012, median follow-up: 2.17 years) suggested a sample size of 203 was needed to obtain the alpha of 0.05 and beta of 0.25. Therefore, all 70 cases from another centre were added to the validation set to make a total of 204. This validation set was only used for testing the findings from the training set. We used the maximum number of patients in the TCIA database that were available at the time. The original number of patients screened and basis for exclusion are indicated in Supplementary Table 1.

Pre-therapy clinico-pathologic data were obtained from medical records (Table 1). Overall survival was defined as number of months from commencement of treatment to date of death. Patients who were alive were censored at last follow-up to 31st July 2016. The hospital records were used to determine who was still alive at the time of cut-off. This was a multi-institutional analysis and so patients were examined on different PET/CT scanners including Phillips Allegro Body, Phillips Gemini TF TOF 16 (Phillips Medical Systems, Amsterdam, Netherlands), Siemens Biograph 64 mCT, Siemens Biograph 128 mCT (Siemens Healthcare, Erlangen, Germany), GE Healthcare Discovery ST, GE Discovery STE (GE Healthcare, Waukesha, Wisconsin, USA), CTI ECAT HR+ (CTI PET Systems Inc., Knoxville, Tennessee, USA), and CPS/Siemens Sensation 16. For PET, slice thickness ranged between 2 and 5.15 mm; the matrix size ranged between 128^2^ and 512^2^. After injection of 350–500 MBq ^18^F-FDG [9], emission data were acquired (five or six bed positions, 2–4 min per bed position) after a 60–90 min uptake period. In all cases, PET/CT scans were performed from upper thighs to the base of the skull following ≥ 4–6-h fast, and had a measured blood glucose level < 11.0 mmol/l at the time of injection. CT was acquired without oral or intravenous contrast agent. The PET data were reconstructed using OSEM iterative reconstruction and were attenuation-corrected using the CT data.

**Table 1:** Characteristics of the training, validation and test datasets

	Training set	Validation set	Test set I
Number	133	204	21
Mean age (range) years	69 (35–89)	71 (42–91)	71 (53–101)
Male (%)	82 (61.7)	126 (61.7)	10 (47.6)
Stage I (%)	24 (18)	33 (16.2)	4 (19)
Stage II (%)	34 (25.6)	37 (18.1)	4 (19)
Stage III (%)	75 (56.4)	134 (65.7)	13 (61.9)
Histology: SCC (%)	69 (51.9)	95 (46.7)	14 (66.7)
Histology: adeno(%)	41 (30.8)	77 (37.7)	5 (23.8)
Histology: NSCLC *NOS* (%)	18 (13.5)	25 (12.3)	2 (9.5)
Histology: other (%)	5 (3.8)	7 (3.4)	0
SUVmean (range)	8.25 (1.78–17.4)	8.44 (2.11–23.7)	7.75 (4.44–16.8)
SUVmax (range)	16.5 (4.9–42.8)	15.9 (3.26–49.5)	13.6 (6.66–39.2)
SUVpeak (range)	14.2 (3.8–35.4)	14.2 (2.9–43.1)	12.5 (6.26–34)
MTV (range) mls	40.4 (5.13–467)	33.7 (5.27–525)	30.8 (7.03–230)
TLG (range)	344 (16.2–5.45 × 10^3^)	315.2 (19.4–5.7 × 10^3^)	266 (40.5–2.59 × 10^3^)
Median overall survival (months)	25 (0–83)	21.0 (0–85)	20 (2–37)
Number of deaths (%)	88 (66.2)	145 (71.1)	17 (81%)
Length of follow-up (median + IQR in months)	26 (12–39)	22.0 (11–36)	21 (8–31)

*SCC* squamous cell carcinoma, *Adeno* adenocarcinoma, *NSCLC* non-small-cell lung cancer (not otherwise specified, i.e., not classified into squamous or adenocarcinoma), *MTV* metabolic tumour volume, *TLG* total lesion glycolysis, *IQR* interquartile range. Stage AJCC/UICC 7

### PET analysis

Central analyses of all PET/CT data were conducted at Imperial College London by a semi-automated adaptive threshold method. The primary tumor was initially delineated using an initial threshold of 40% of the SUV_max_ on semi-automated software (Hermes Gold3; Hermes Medical Solutions Ltd., London, UK) and VOIs drawn. The PET volume was correlated with the primary tumor on CT, and underestimation was determined by checking if the PET tumour VOI encompassed the whole tumour on the CT component of the PET. If the VOI did not cover the tumour visually, a lower threshold was used [10]. Manual adjustment was employed when the VOI incorporated adjacent normal structures such as adjacent myocardial activity [11]. All segmentations were made by the same operator (observer 1, a radionuclide specialist radiologist with 4 years’ experience of tumor delineation).

The SUV_mean_, SUV_max_, SUV_peak_, metabolic tumor volume (MTV), and total lesion glycolysis (SUV_mean_ × MTV)(TLG) of the primary tumor were recorded. Using Youdens’s J to find the optimal cut-off from the ROC for median survival, Kaplan–Meier curves were generated. The VOIs were extracted and imported into the radiomics software. To assess intra- and inter-observer variability of the segmentation method, 18 patients were selected at random by SPSS, and segmentation of the tumor was performed (at 128 Gy level) by two additional experienced operators (observers 2 and 3, with 6 and 10 years’ experience of tumor delineation respectively) blinded to the original results and clinical data. Lymph nodes were excluded from statistical analyses.

The interclass coefficient was used to assess intra- (by observer 1) and inter-observer (by observers 1, 2, and 3) differences in texture. The differences between the observers were performed by a 2-way ANOVA repeated measures model using Bonferroni correction.

### Radiomics analysis

Radiomics analysis (Supplementary Fig. 1) was performed at seven different quantisation/gray levels — 4, 8, 16, 32, 64,128 and 256 Gy — on TexLAB v2, which was developed and implemented in-house within Matlab R2015b (MathWorks Inc., Natick, MA, USA). From each primary tumor, 665 radiomic features (listed in Supplementary Table 3) were extracted from segmented VOIs using local, regional, global, fractal, and wavelet techniques. These included intensity features, shape features, and texture features [gray level co-occurrence matrix (GLCM), gray level run length matrix (GLRLM) and neighbourhood gray difference matrix (NGTDM)] with or without wavelet transformation, as previously reported [5, 6]. Radiomics features were determined from 133 PET scans (training set) using TexLAB *v2*.

### Feature selection and radiomics signature discovery

As with other high-throughput analyses, it is important to reduce the total number of features for prediction purposes in order to eliminate Type 1 errors and instead learn the true basis of a decision. We initially identified highly correlated features for elimination using heatmaps, as highly correlated features suggested that some feature reduction could be undertaken without information being lost. Heatmaps were created using R software (http://www.r-project.org/; Version 3.03 Vienna, Austria). It is known that there is correlation of several texture features with volume [12]. Using Spearman's rank correlation, features that had a high correlation with volume (≥ 0.7) were normalised by dividing the feature value by volume to obtain volume-invariant texture features (notably, the two features included in the final analysis did not correlate with volume, and thus, did not require normalisation to volume).

From the 665 sets of features at each gray-level, we used least absolute shrinkage and selection operator (LASSO) regression analysis for data dimension reduction, radiomics feature vector (composite feature) discovery, generating Kaplan–Meier curves and computing the Cox regression analysis. LASSO is a form of penalised regression used to reduce the problem of multi-collinearity. Briefly, the non-contributory variables were assigned zero-weighting, and numerous iterations were performed to link the non-zero contributory variables to the chosen clinical outcome (in this example, overall survival) [13]. Analyses were conducted with R software; the packages in R used for our analysis are indicated in Supplementary Table 4. Two-sided statistical significance levels were used, and *p* ≤ 0.05 was considered statistically significant. SPSS for Statistics Version 22 (IBM, Armonk, NY, USA) was used for interclass correlation and 2-way ANOVA.

The most predictive feature vectors (FVX) were computed by linear combination of selected statistical features of the matrices weighted by their respective coefficients and by comparison with overall survival (OS). Survival curves were plotted using Kaplan–Meier (KM) methods, stage-specific or Youden’s J cut-off on the receiver operator curve for the median survival in the case of FVX. Kaplan–Meier curves were plotted for overall survival using the ‘survfit’ function from the ‘survival’ package in R using the median cut-off for the MTV, TLG, and FVX. The statistical significance of the difference in the survival curves was calculated using the logrank test implemented in the ‘survdiff’ function. The survival curves were evaluated using a log-rank test (Cox Regression). Multivariable analysis of the FVX, stage, MTV, and TLG were compared with each other using a stepwise backward procedure to determine significantly independent survival indicators. *P* values of ≤ 0.05 were considered statistically significant, and 95% confidence intervals were calculated. A continuous Cox regression and the C-index, was computed for each prognosticator in the univariate analysis, and for the multivariable analysis with and without FVX. All four variables (FVX, stage, MTV, and TLG) were used as continuous variables in the analysis.

### Independent validation and testing

Performance of the FVX and stage were tested by comparison to OS in an independent validation set of 204 patients, and a further independent set of 21 (TESTI; the final institutional dataset to be accepted into the study) patients. Similar survival comparisons were made with routine PET variables including SUV_mean_, SUV_max_, SUV_peak_, MTV, and TLG.

## Results

### Patient characteristics and PET analysis

Patient characteristics are displayed in Table 1. There were no significant differences in the proportion of males to females except in TESTI, which was a very small dataset. The majority of patients were, as expected for such a cohort, stage III. Typical PET images are shown in Fig. 1b. Primary tumor SUV_max_ ranged from 3.3 to 49.5 (Table 1). All patients were treated with radiotherapy with or without chemotherapy, and median survival values were not significantly different between the training, validation, and independent test cohorts, except in the small cohort in TESTI. No cases treated primarily by surgery were analysed, as the inclusion criterion was the cohort having radiotherapy with or without chemotherapy rather than surgery. Admittedly, some patients with stage I or II disease would have been unfit for surgery, while others would have elected for radiotherapy with or without chemotherapy in preference to surgery due to factors including patient choice; we do not have accurate data for the reasons for this choice. Of 358 patients, 249 died within the follow-up period [median 22 (range 0–85) months]). The comprehensive data for scanners are provided in Supplementary Table 2.

Segmentation is an important source of variability in radiomics analysis [14]. The most prevalent threshold cut-off values were 40% (47.8% of cases) and 30% (27.7% of cases); together these accounted for 75.5% of all the thresholds; 24.5% required a lower threshold value in order to encompass the whole tumour as defined by the CT component of the PET. Furthermore, 9.5% of cases required additional manual adjustment, after setting the initial threshold, to achieve optimal segmentation. Intra- and inter-observer variability of radiomics features from 18 randomly selected patients are displayed in Supplementary Tables 5 and 6. There was near-perfect [15, 16] intra- and inter-observer variability in the PET-derived radiomics features. The intra-observer variability of the radiomics features alone and when combined with PET features was 0.9 and 0.92 respectively. Corresponding inter-observer variability values were 0.86 and 0.88 respectively.

### Radiomics feature vector selection

**Fig. 2:**
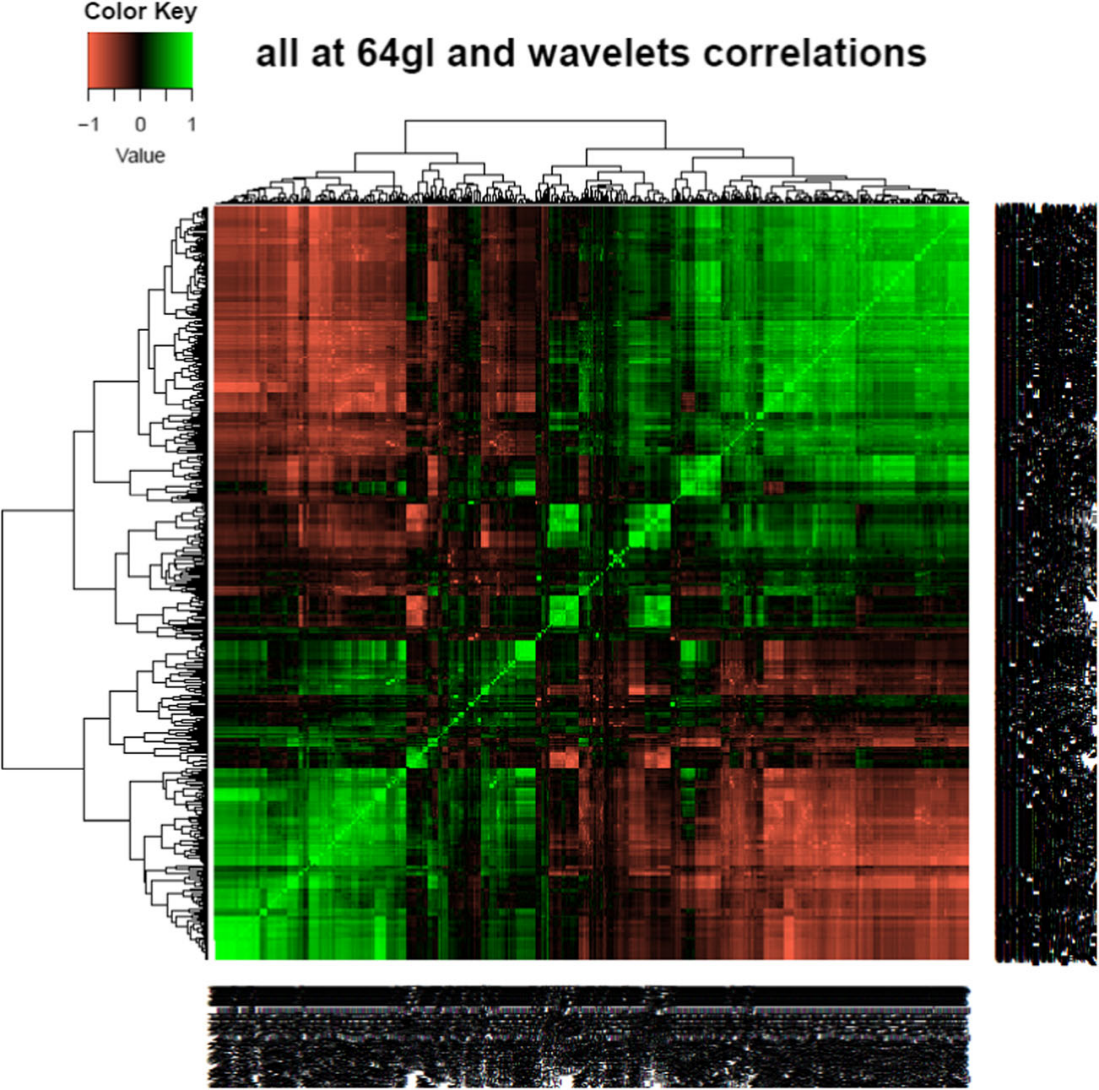
Spearman rank correlation of the radiomics features displayed as a heatmap. High-level correlation with clustering of features is seen

Radiomics predictive classifiers (665) were derived from TexLAB *v2*. Generation of a heatmap (Fig. 2) from all the patient data — both training and validation sets — visually indicated multi-collinearity (when many features are related), and suggested that some features could be reduced without information being lost. From the 665 radiomics features returned by the software in the training set of 133 patient scans, one FV was selected as the optimal predictor (FVX) — a weighted linear combination of the statistical features of size-variance of the gray-level size zone matrix at 64 Gy levels (GLSZM_SzVarianc_64gl; weighted by 0.128) and complexity of the neighbourhood gray-tone difference matrix (NGTDM) at 64 Gy levels (NGTDM_Complex_64gl; weighted by −0.018) — using LASSO conducted independently at each gray level (example shown in Supplementary Fig. 2). Previous studies have indicated that primary tumor volume is an important predictor of survival in lung cancer [17]. A multivariable analysis was performed which included the new FVX, volume (SNS_vol_ variable) and stage; volume was not significant, and was not further considered. Both the stage and FVX were significant and, most importantly, there was no correlation between the two (*p* = 0.22).

**Fig. 3:**
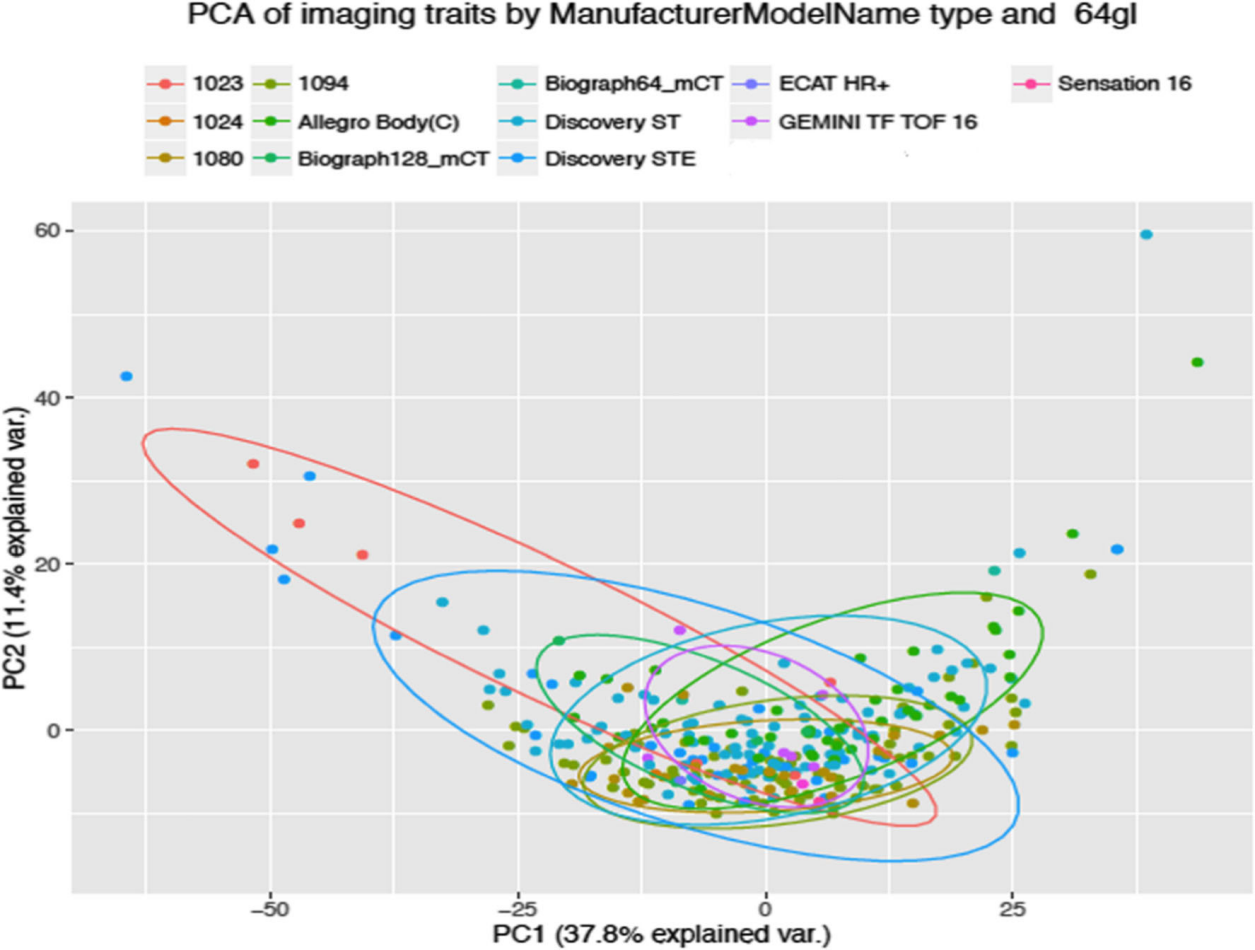
Principal component analysis (explained variance) of PET radiomics features (at 64 Gy level) to assess congruence of data from different manufacturer models: CPS 1023, CPS 1024, Siemens 1080, 1094, Phillips Allegro Body (C), Siemens Biograph 64 mCT, Siemens Biograph 128 mCT, GE Discovery ST, GE Discovery STE, CTI ECAT HR+, Phillips Gemini TF TOF 16, and CPS/Siemens Sensation 16 respectively

We tested the influence of PET scanner equipment properties on the FVX. Principal component analysis of FVX (at 64 Gy levels) was used to assess the congruence of data across different manufacturer types, manufacturer models, slice thickness, number of rows, or number of columns (Fig. 3; Supplementary Figs. 3–5 and Supplementary Table 7). All elements of the data were tightly clustered around each other (minimal variance), suggesting that FVX was invariant to the type of PET/CT manufacturer or slice thickness; thus, no correction was made for sets of data from different institutions. Other FVs were dependent on scanner type (*data not shown*).

### Performance of radiomics feature vector

**Fig. 4:**
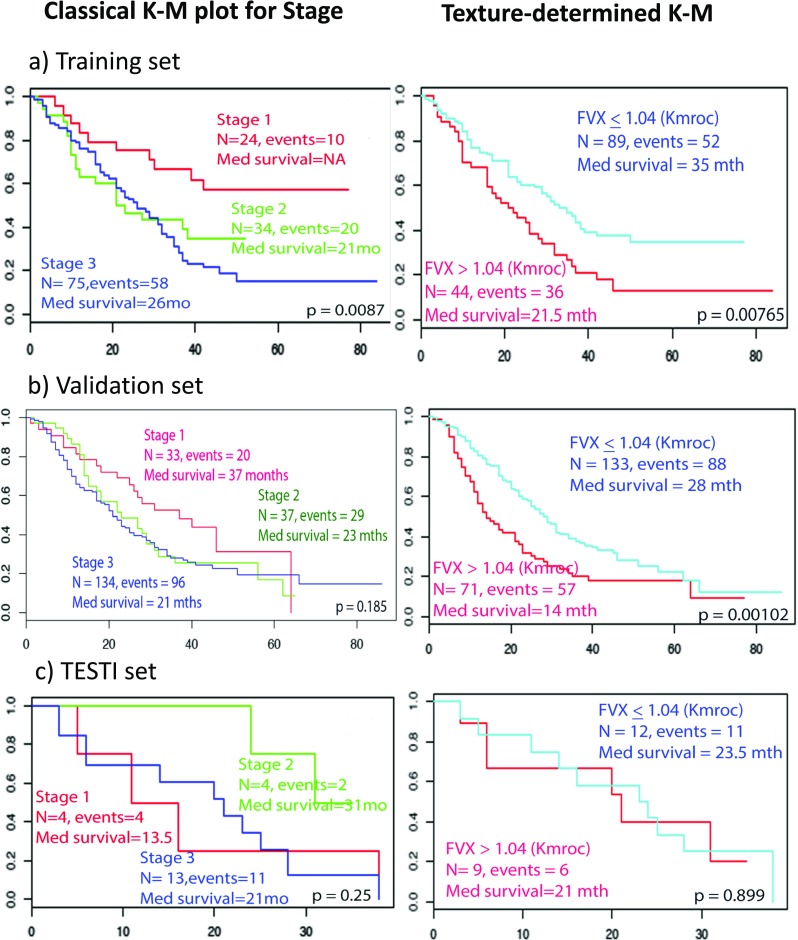
Survival analysis based on composite radiomics feature dichotomized using ROC. Kaplan–Meier plots of **a** training, **b** independent validation, and **b** TESTI. Note that the validation dataset has longer follow-up period. *K–M *= Kaplan–Meier, *N* = number of subjects, *mths, mo, mth* = months, *Med *= median

**Fig. 5:**
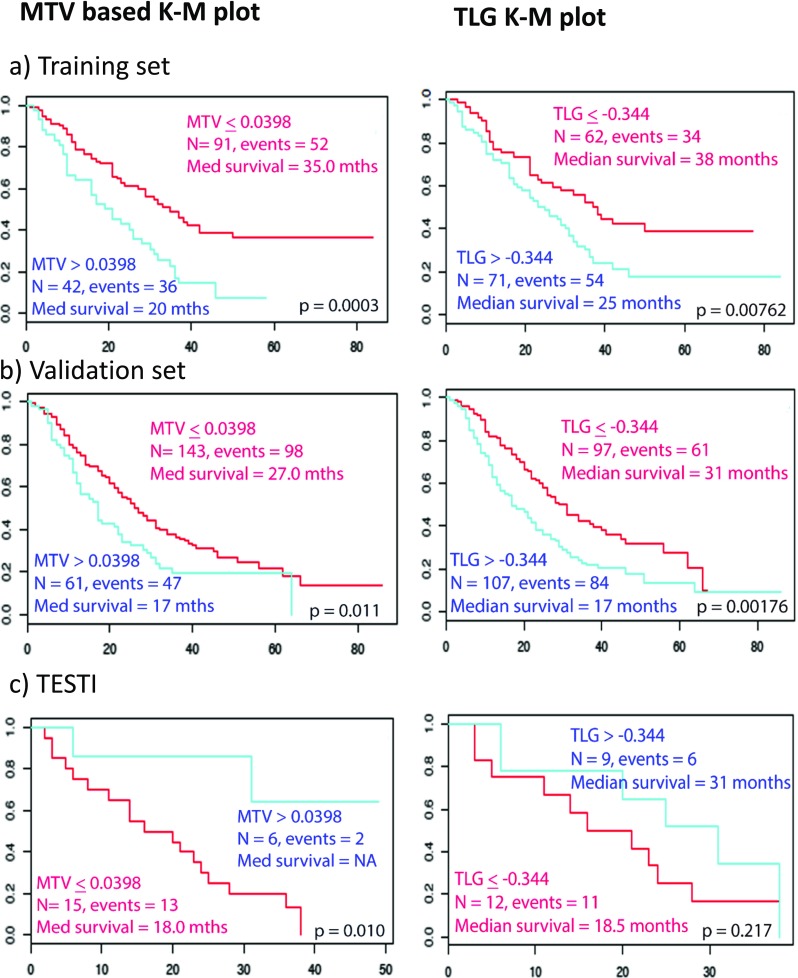
Survival analysis based on the SUV variables, MTV and TLG, dichotomized using ROC. Kaplan–Meier plots of **a** training dataset, **b** independent validation set, and **c** independent TESTI. Note that the validation dataset has a longer follow-up period. *MTV* metabolic tumour volume, *TLG* total lesion glycolysis

We tested the performance of FVX in an independent validation cohort comprised of 204 patient scans by comparison to OS. Kaplan–Meier (KM) plots for stage and FVX are shown in Fig. 4. FVX was significantly associated with OS in the validation set when dichotomised at median (*p* = 0.00465), optimal cut-off by Kmroc (*p* = 0.00116) or as a continuous variable (*p* = 0.00429), with hazard ratios (HRs) of 1.61 (1.16–2.24), 1.74 (1.25–2.44), and 5.30 (1.69–16.6) respectively. In the TESTI cohort that presented with an unusually poor survival of the four stage I cancers (Supplementary Figs. 6 and 7), FVX correctly indicated a lack of survival difference (*p* = 0.501). FVX values for image data presented in Fig. 1b, for instance, were − 29.9 and − 03.1 for patients 1 and 2 respectively, thus correctly predicting survival relative to stage. In contrast to the radiomics classifier, clinically routine PET variables including SUV_max_, SUV_mean_, and SUV_peak_ lacked any predictive information (Supplementary Fig. 8). The MTV and TLG were significant on the KM plots; surprisingly, MTV was also significant on the TESTI KM plot (Fig. 5). The MTV and TLG were highly correlated with each other (Supplementary Table 8), but neither the TLG nor MTV when tested separately with the FVX and stage were significant on the multivariable Cox regression (Supplementary Table 9).

FVX, stage, MTV, and TLG were the only potential prognosticators that showed significance in the univariate analysis. The Cox regression analysis associating FVX, stage, MTV, and TLG with overall survival in three datasets are summarised in Supplementary Table 9, with both univariate analysis and multivariable analysis combining all the four variables. FVX was prognostic, independent of stage, MTV, and TLG in both training and validation sets. MTV and TLG were not significant once combined with FVX in the multivariable model, suggesting that FVX is a significantly better prognosticator than MTV and TLG.

## Discussion

This multi-institution retrospective study showed that a radiomics feature vector, FVX, derived from analysis of FDG-PET data of primary tumors ≥ 5 ml in patients with NSCLC is invariant to PET scanner properties and predicts OS. Accurate prognostic information is crucial in stratifying newly diagnosed NSCLC patients to different treatments or best supportive care. Currently, TNM staging is the primary method to stratify treatment approach in NSCLC; however, it offers imprecise prognostic information, leading to both under-treatment and over-treatment in some patients. Other established prognostic factors for lung cancer include performance status (Karnofsky or ECOG (Eastern Cooperative Oncology Group) classification), weight loss (e.g., > 5%) and systemic inflammation (C-reactive protein or modified Glasgow Prognostic Score) [18–20]. While factors such as EGFR (epidermal growth factor receptor) mutation predict response to targeted therapy [21–23], tumor-specific prognostic factors are lacking. In the current work, we assessed the role of radiomics features as prognostic factors in NSCLC. A machine-learning-enabled weighted linear combination of the statistical features of GLSZM_SzVarianc_64gl and NGTDM_Complex_64gl — FVX — was found to possess prognostic information and importantly was invariant to scanner properties investigated (Supplementary Table 7). The features do not have immediate physiological relevance. GLSZM is a regional ‘homogeneity’ texture feature that calculates lengths of uniform pixels (picture elements) in a 2D image, or in our case, directionally-independent groups of uniform voxels (volume elements) in each of the 26 available directions in 3D space; GLSZM_SzVarianc_64gl (size variance of the GLSZM at 64 Gy level) examines the variance in the number blocks by size (independent of the gray-level) and is negatively correlated with survival, possibly identifying hypoxic or necrotic regions with poor prognosis [24–26]. NGTDM represents contrast, and is determined by examining changes in intensity between a target voxel and the surrounding neighbors to enable calculation of apparent difference between neighboring regions of voxel intensities. Contrast is related to the information content of an image and is a mathematical measure of heterogeneity; non-responding tumors with poor prognosis tend to have higher contrast [27]. NGTDM_Complex_64gl (complexity of the NGTDM for 64gl), which refers to the average visual complexity within the volume, is positively correlated with survival, although with less of a magnitude than SzVarianc, and perhaps acting as a balance on SzVarianc.

This is one of the first reports of a whole tumor image-derived lung cancer prognostic factor. In our analysis, there was a higher hazard of death (1.74; *p* = 0.00116) when the median FVX was used as input. The implicit assumption here is that a set of mathematically-derived tumor phenotypes correlate with survival. It should be noted, however, that death as an endpoint could have occurred by other means — indirect consequence of the tumor or non-tumor related — or may have been subjected to different variations of physician choice of chemotherapy/chemo-radiotherapy, making this analysis the more interesting. Furthermore, routine PET variables, while useful for staging, did not possess prognostic information. Baseline primary tumor SUV_max_ has been reported by some groups, but not all, to predict outcome in NSCLC patients. For resectable NSCLC patients, a meta-analysis of 13 studies showed that primary tumor SUV_max_ has significant prognostic value on patient survival [28]. A more recent meta-analysis, assessing the prognostic value of primary tumor SUV_max_ prior to radiotherapy in NSCLC, reported that higher tumor SUV_max_ was correlated with shorter OS, particularly in stage I NSCLC receiving stereotactic body radiotherapy (SBRT) [29].

Volumetric parameters, such as MTV and TLG, which consider the whole tumor volume, have been reported to be prognostic in NSCLC. Secondary analysis of the large multicentre prospective American trial of 196 inoperable Stage IIb/III NSCLC has reported MTV, TLG to be strongly prognostic for OS, while SUV_max_ was not [30]. The TLG and MTV, which were shown in our study to highly correlate with each other, were not significant on multivariable analysis.

As this was a large multi-centre study acquired on different scanners, voxel sizes differed (Supplementary Table 2); we did not standardise the voxel sizes. However, slice thickness and matrix size did not significantly affect the FVX. The robustness of FVX, invariant to instrument factors including slice thickness, permits this variable to be applied in multi-institutional studies. Previous work on scanner types [31–33] have yielded mixed results in terms of texture stability across model and manufacturer type, although limited models have been used. In addition, much thought has been given in the methodology to reduce Type 1 errors and false discovery which have entered the published literature [34]. Compared to CT technology that has seen substantive reduction in slice thickness, PET FWHM (full width at half-maximum; a measure of resolution) has not seen such substantive change over the past decade, and this could have led to the scanner invariance of our study. We set a threshold of 5 ml, in keeping with earlier work of Soussan [8]. It is likely that inclusion of smaller tumors would have led to higher variability given the poor resolution of PET (compared to CT or MRI). Hence, the inferences from this study are limited to the group of patients presenting with medium-large lesions. The classification of patient subgroups in the Kaplan–Meier analysis was based on the FVX value calculated from combination of weighted radiomic features. FVX was a continuous variable, and we have demonstrated that FVX was linearly correlated with overall survival in the training and validation sets using a continuous Cox regression analysis. It should be noted that other analyses were performed: Cox regression based on dichotomised median FVX, Kmeans clustering and optimal cut-off (Youden’s J) from the ROC curve, and showed consistent results (data not shown).

TESTI was an unusual dataset in terms of size and heterogeneity with a high number of stage 1 tumors and high mortality. The fact that these patients had radiotherapy ± chemotherapy instead of surgery indicates that there was probably associated poor performance status. Unfortunately, information on performance status was not available. However it was felt important to attempt to test the radiomics signature against this unusual dataset, as ideally the ‘real-life’ prediction of the radiomics feature vector should work irrespective of sample size.

A recent single-institution study of a PET/CT radiomics signature for prediction of disease-free survival (DFS) in NSCLC undergoing surgery with curative intent reported that image derived parameters outperformed TNM staging in predicting DFS. However, although promising, this was a single-centre study, utilising what appears to be unenhanced CT scans without external validation and in a different cohort of patients to our study [35].

Limitations of the present study should be highlighted. 1) This is a retrospective, albeit multi-institutional, study and future prospective studies in similarly large cohorts will be needed to verify this novel endpoint. 2) We did not consider other prognostic factors as these were not consistently available from all institutions. Addition of other prognostic factors in future studies will enable more rigorous assessment of events likely to have caused death. 3) We did not consider EGFR mutations/expression or other genetic outcomes, as these were not available for all patients. 4) Approximately 14% of the initial 535 patients screened were excluded for having tumours < 5 ml (Supplementary Table 1). The choice of 5 ml reflects a statistical limitation of applying radiomics to PET data (less data-points within the VOI compared to CT). Thus, with regard to generalisation of our study to patients having chemo-radiotherapy, we would caution the exploitation of our findings to smaller tumors. Irrespective of these limitations, we highlight a massive opportunity for physicians and patients, whereby mathematically-derived features from scans that newly diagnosed NSCLC patients would normally have as part of routine care can be ‘re-purposed’ to predict prognosis. Only software implementation and computing power are required for incorporation into patient management pathways; thus, we envisage easy acceptance of this potentially cost-effective methodology for use with existing prognostic methods. As it is tumor-specific, patients stratified to poor prognostic FVX groups could be candidates for earlier follow-up or a lower threshold in change of therapy [36].

In summary, we have discovered a scanner-invariant radiomics feature vector that performs well in independent validation and test datasets. This multi-institutional study provides new opportunities for prospective assessment of radiomics features for prognosis in patients with NSCLC.

## Electronic supplementary material

ESM 1 (PDF 3235 kb)

## References

[1] Jemal A, Bray F, Center MM, Ferlay J, Ward E, Forman D (2011). Global cancer statistics. CA Cancer J Clin.

[2] de Bruin EC, McGranahan N, Mitter R, Salm M, Wedge DC, Yates L (2014). Spatial and temporal diversity in genomic instability processes defines lung cancer evolution. Science.

[3] Zhang J, Fujimoto J, Zhang J, Wedge DC, Song X, Zhang J (2014). Intratumor heterogeneity in localized lung adenocarcinomas delineated by multiregion sequencing. Science.

[4] Segal E, Sirlin CB, Ooi C, Adler AS, Gollub J, Chen X (2007). Decoding global gene expression programs in liver cancer by noninvasive imaging. Nat Biotechnol.

[5] Aerts HJ, Velazquez ER, Leijenaar RT, Parmar C, Grossmann P, Cavalho S (2014). Decoding tumour phenotype by noninvasive imaging using a quantitative radiomics approach. Nat Commun.

[6] Willaime JM, Turkheimer FE, Kenny LM, Aboagye EO (2013). Quantification of intra-tumour cell proliferation heterogeneity using imaging descriptors of 18F fluorothymidine-positron emission tomography. Phys Med Biol.

[7] Wu W, Parmar C, Grossmann P, Quackenbush J, Lambin P, Bussink J (2016). Exploratory study to identify radiomics classifiers for lung cancer histology. Front Oncol.

[8] Soussan M, Orlhac F, Boubaya M, Zelek L, Ziol M, Eder V (2014). Relationship between tumor heterogeneity measured on FDG-PET/CT and pathological prognostic factors in invasive breast cancer. PLoS One.

[9] Boellaard R, Delgado-Bolton R, Oyen WJG, Giammarile F, Tatsch K, Eschner W (2015). FDG PET/CT: EANM procedure guidelines for tumour imaging: version 2.0. Eur J Nucl Med Mol Imaging.

[10] Biehl KJ, Kong FM, Dehdashti F, Jin JY, Mutic S, El Naqa I (2006). 18F-FDG PET definition of gross tumor volume for radiotherapy of non-small cell lung cancer: is a single standardized uptake value threshold approach appropriate?. J Nucl Med.

[11] Foster B, Bagci U, Mansoor A, Xu Z, Mollura DJ (2014). A review on segmentation of positron emission tomography images. Comput Biol Med.

[12] Brooks FJ, Grigsby PW (2013). FDG uptake heterogeneity in FIGO IIb cervical carcinoma does not predict pelvic lymph node involvement. Radiat Oncol.

[13] Tibshirani R (1997). The lasso method for variable selection in the Cox model. Stat Med.

[14] Leijenaar RT, Nalbantov G, Carvalho S, van Elmpt WJ, Troost EG, Boellaard R (2015). The effect of SUV discretization in quantitative FDG-PET radiomics: the need for standardized methodology in tumor texture analysis. Sci Rep.

[15] Büyükdereli G, Güler M, Şeydaoğlu G (2016). Interobserver and Intraobserver variability among measurements of FDG PET/CT parameters in pulmonary tumors. Balkan Med J.

[16] Armitage P, Berry G, Matthews JNS (2001). Statistical methods in medical research.

[17] Ball DL, Fisher RJ, Burmeister BH, Poulsen MG, Graham PH, Penniment MG (2013). The complex relationship between lung tumor volume and survival in patients with non-small cell lung cancer treated by definitive radiotherapy: a prospective, observational prognostic factor study of the Trans-Tasman Radiation Oncology Group (TROG 99.05). Radiother Oncol.

[18] Lee ES, Son DS, Kim SH, Lee J, Jo J, Han J (2008). Prediction of recurrence-free survival in postoperative non-small cell lung cancer patients by using an integrated model of clinical information and gene expression. Clinical Cancer Res.

[19] Detterbeck FC, Postmus PE, Tanoue LT (2013). The stage classification of lung cancer: diagnosis and management of lung cancer, 3rd ed: American College of Chest Physicians evidence-based clinical practice guidelines. Chest.

[20] Alberg AJ, Brock MV, Ford JG, Samet JM, Spivack SD (2013). Epidemiology of lung cancer: diagnosis and management of lung cancer, 3rd ed: American College of Chest Physicians evidence-based clinical practice guidelines. Chest.

[21] Majem M, Remon J (2013). Tumor heterogeneity: evolution through space and time in EGFR mutant non small cell lung cancer patients. Transl Lung Cancer Res.

[22] Nana-Sinkam SP, Powell CA (2013). Molecular biology of lung cancer: diagnosis and management of lung cancer, 3rd ed: American College of Chest Physicians evidence-based clinical practice guidelines. Chest.

[23] Chen Z, Fillmore CM, Hammerman PS, Kim CF, Wong KK (2014). Non-small-cell lung cancers: a heterogeneous set of diseases. Nat Rev Cancer.

[24] Overgaard J (2007). Hypoxic radiosensitization: adored and ignored. J Clin Oncol.

[25] Wilson WR, Hay MP (2011). Targeting hypoxia in cancer therapy. Nat Rev Cancer.

[26] Thibault G, Fertil B, Navarro C, Pereira S, Cau P, Levy N (2009). Texture indexes and gray level size zone matrix application to cell nuclei classification. PRIP.

[27] Sun CJ, Wee WG (1983). Neighboring gray level dependence matrix for texture classification. Comput Vision Graph.

[28] Berghmans T, Dusart M, Paesmans M, Hossein-Foucher C, Buvat I, Castaigne C (2008). Primary tumor standardized uptake value (SUVmax) measured on fluorodeoxyglucose positron emission tomography (FDG-PET) is of prognostic value for survival in non-small cell lung cancer (NSCLC): a systematic review and meta-analysis (MA) by the European Lung Cancer Working Party for the IASLC Lung Cancer Staging Project. J Thorac Oncol.

[29] Na F, Wang J, Li C, Deng L, Xue J, Lu Y (2014). Primary tumor standardized uptake value measured on F18-Fluorodeoxyglucose positron emission tomography is of prediction value for survival and local control in non-small-cell lung cancer receiving radiotherapy: meta-analysis. J Thorac Oncol.

[30] Salavati A, Duan F, Snyder BS, Wei B, Houshmand S, Khiewvan B, et al. Optimal FDG PET/CT volumetric parameters for risk stratification in patients with locally advanced non-small cell lung cancer: results from the ACRIN 6668/RTOG 0235 trial. Eur J Nucl Med Mol Imaging. 2017; 44(12):1969–83 10.1007/s00259-017-3753-x PMC5648620 28689281

[31] Reuzé S, Orlhac F, Chargari C, Nioche C, Limkin E, Riet F (2017). Prediction of cervical cancer recurrence using textural features extracted from (18)F-FDG PET images acquired with different scanners. Oncotarget.

[32] Galavis PE, Hollensen C, Jallow N, Paliwal B, Jeraj R (2010). Variability of textural features in FDG PET images due to different acquisition modes and reconstruction parameters. Acta Oncol.

[33] Parmar C, Rios Velazquez E, Leijenaar R, Jermoumi M, Carvalho S, Mak RH (2014). Robust radiomics feature quantification using semiautomatic volumetric segmentation. PLoS One.

[34] Chalkidou A, O'Doherty MJ, Marsden PK (2015). False discovery rates in PET and CT studies with texture features: a systematic review. PLoS One.

[35] Kirienko M, Cozzi L, Antunovic L, Lozza L, Fogliata A, Voulaz E (2018). Prediction of disease-free survival by the PET/CT radiomic signature in non-small cell lung cancer patients undergoing surgery. Eur J Nucl Med Mol Imaging.

[36] Okimoto RA, Bivona TG (2014). Recent advances in personalized lung cancer medicine. Pers Med.

