# Supplementary material for: Discovery of pre-therapy 2-deoxy-2-18F-fluoro-D-glucose positron emission tomography-based radiomics classifiers of survival outcome in non-small-cell lung cancer patients
Source: Eur J Nucl Med Mol Imaging. 2018 Sep 1;46(2):455–66. doi: 10.1007/s00259-018-4139-4 (PMC6333728; doi:10.1007/s00259-018-4139-4)
Supplement: Supplementary file 1 — (PDF 3235 kb) [file 259_2018_4139_MOESM1_ESM.pdf]

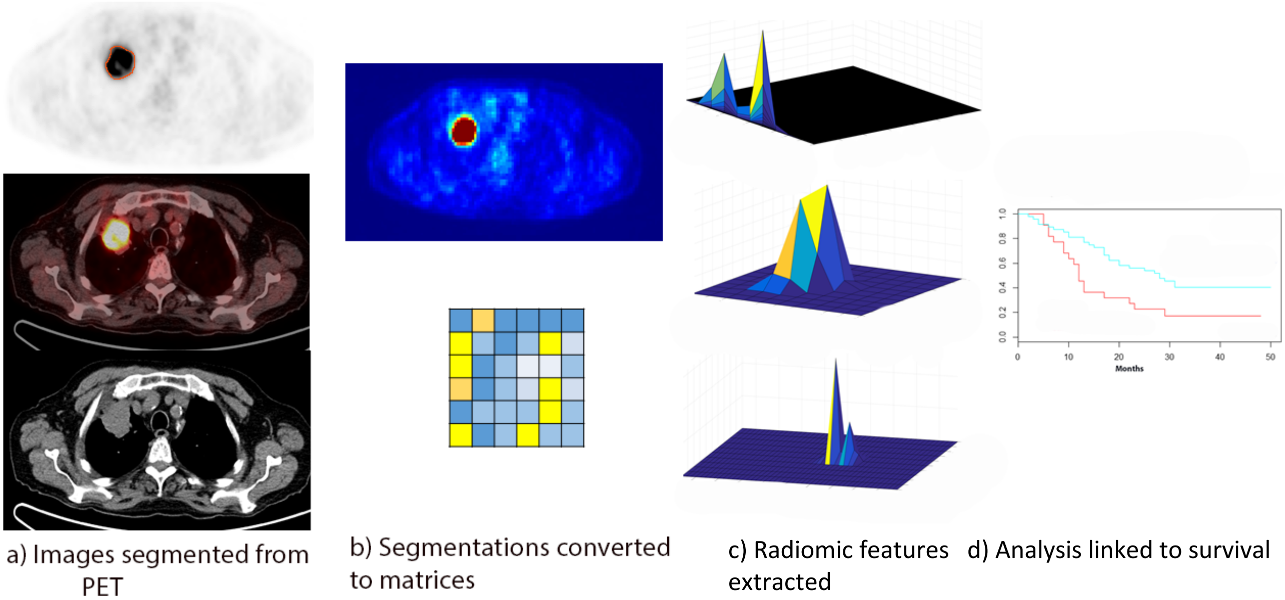

Supplementary Figure 1 Overview of the radiomics analysis process: a) The images are obtained from PET and are segmented, b) the imaging analysis software utilises the segmentation to develop different matrices, c) the output for various radiomics features at different grey levels is obtained, d) these are linked with clinical features such as survival.

Supplementary Table 1: Original number of patients screened from different centres and basis for exclusion of scans.

	Imperial	Kings	Leeds	Marsden	TCIA	Mount Vernon	Nottingham	Total
Original number	112	53	105	91	29	102	43	535
Wrong histology	12					4		16
Metastatic	1		13		1	3		18
Too small	20	4	27	10	6	9	1	77
No scan available		1		22			15	38
Primary not visualised			2	4		5		11
No clinical details						11		11
Different treatment							6	6
Total excluded	33	5	42	36	7	32	22	177
Total included	79	48	63	55	22	70	21	358

Supplementary Table 2. Description of scanner properties used in this study indicating manufacturers, models, slice thickness and matrix size from the different centres.

	Imperial (%)	KCL (%)	Leeds (%)	TCIA (%)	Marsden (%)	Nottingham (%)	Mount Vernon (%)	All (%)
**Number**	79	48	63	22	55	21	70	358
**Manufacturer**								
CPS				8 (36.4)				8 (2.2)
CTI				1 (4.5)				1 (0.3)
GE medical systems		48 (100)	63 (100)	10 (45.5)			67 (95.7)	188 (52.5)
Phillips Medical Systems					50 (90.1)		1 (1.4)	51 (14.2)
Siemens	79 (100)			3 (13.6)	5 (9.09)	21 (100)	2 (2.9)	110 (30.7)
**Model**								
CPS 1023				7 (31.8)				7 (2.0)
CPS 1024				1 (4.5)				1 (0.3)
Siemens 1080				3 (13.6)		21 (100)		24(6.7)
Phillips Allegro Body (C)					44 (80)			44 (12.3)
Siemens Biograph128 mCT					4 (7.3)			4 (1.1)
Siemens Biograph64	79 (100)						1 (1.4)	80 (22.3)
Siemens Biograph64 mCT					3 (5.5)		1 (1.4)	4 (1.1)
GE Discovery ST		24 (50%)	58 (92.1)	5 (22.7)			30 (42.9)	117 (32.7)
GE Discovery STE		24 (50)	5 (7.9)	5 (22.7)			37 (52.9)	116 (32.4)
CTI ESCAT HR+				1 (4.5)				1 (0.3)
Phillips GEMINI TF TOF 16					4 (7.3)			4 (1.1)
Phillips GEMINI TF TOF 64							1 (1.4)	1 (0.3)
**Matrix**								
128 x 128	79 (100)	48 (100)	63 (63)	19 (86.4)			67 (95.7)	276 (77.1)
144 x 144					49 (89)		1 (1.4)	50 (14.0)
168 x 168				3 (13.6)		21(100)	1 (1.4)	25 (7.0)
169 x 169					1 (1.8)			1 (0.3)
200 x 200					5 (9.1)		1 (1.4)	6 (1.7)
**Slice thickness**								
2				2 (9.1)				2 (0.6)
3					7 (12.7)		1 (1.4)	8 (2.2)
3.27		48 (100)	63 (100)	16 (72.3)			67 (95.7)	194 (54.2)
3.38				2 (9.1)				2 (0.6)
4					48 (87.3)		1 (1.4)	49 (13.7)
5	79 (100)			1 (4.5)		21 (100)	1 (1.4)	102 (28.5)
5.15				1 (4.5)				1 (0.3)

Supplementary Table 3. Texture Features-A list of class specific radiomics features used in the study

Technique (Total number)	Features
First order statistics (FOS) (15)	Coefficient of Variation<br>Mean<br>Median<br>Mode<br>Standard Deviation<br>Minimum<br>Maximum<br>Range<br>Skewness<br>Kurtosis<br>Mean Absolute Deviation<br>Root mean square<br>Area under the Curve<br>Entropy<br>Energy
Grey-level co-occurrence matrix (GLCM) (23)	Variance<br>Correlation<br>Information Measure of Correlation 1)<br>Information Measure of Correlation 2)<br>Cluster Shade<br>Cluster Prominence<br>Angular Second Moment<br>Maximum Probability<br>Entropy<br>Contrast<br>Dissimilarity<br>Homogeneity<br>Sum Average<br>Sum Variance<br>Sum Entropy<br>Difference in Variance<br>Difference entropy<br>Autocorrelation<br>Cluster Tendency<br>Homogeneity 1<br>Inverse Difference Moment Normalised<br>Inverse Difference Normalised<br>Inverse Variance

Grey-level size zone matrix (GLSZM) (13)	Small Zone Emphasis<br>Large Zone Emphasis<br>Grey-level Non Uniformity<br>Size zone Non uniformity<br>Zone Percentage<br>Zone Low grey-level Emphasis<br>Zone Low grey-level Emphasis<br>Small Zone Low grey level Zone Emphasis<br>Small Zone High grey level Zone Emphasis<br>Large Zone Low grey level Emphasis<br>Large Zone High grey level Emphasis<br>Grey Level Variance<br>Size-Zone Variance
Neighborhood grey- tone difference matrix (NGTDM) (5)	Coarseness<br>Contrast<br>Busyness<br>Complex<br>Strength
Size and Shape SNS) (8)	Volume<br>Area<br>Surface to volume ratio<br>Sphericity<br>Spherical disproportion<br>Compactness 1<br>Compactness 2<br>Maximum 3d diameter
Grey-Level Run Length Matrix (GLRLM) (11)	Short Run Emphasis<br>Long Run Emphasis<br>Grey-Level Non-Uniformity<br>Run Length Non-Uniformity<br>Run Percentage<br>Low Grey-Level Run Emphasis<br>High Grey Level Run Emphasis<br>Short Run Low Grey Level Emphasis<br>Short Run High Grey Level Emphasis<br>Long Run Low Grey Level Emphasis<br>Long Run High Grey Level Emphasis
Fractal Dimension (FD) (6)	Mean<br>Standard deviation<br>Variance<br>Lacunarity<br>Maximum<br>Minimum
Wavelet transformation x8	As above with 8 filters
Total number of features =665	

Supplementary Table 4. The packages of R software used for statistical analysis

Statistical tool	Packages used
PCA analysis	‘devtools’<br>‘ggbiplot’<br>‘vqv’
Heatmaps with Spearman Correlation	‘gplots’
LASSO binary logistic regression, Kaplan Meier curves, Cox regression	‘doParallel’<br>‘Matrix’<br>‘glmnet 2.0-5’<br>‘survival’

Supplementary Table 5. Overview of intra-observer variability of different radiomics features defined as: 0.2-0.4 fair agreement, 0.4-0.6 moderate, 0.6-0.8 substantial and >0.8 almost perfect agreement. N=18 patients..

Radiomics features	ICC	95% CI
**FOS**	0.97	0.94-0.99
**GLCM**	0.82	0.69-0.90
**GLSZM**	0.93	0.85-0.97
**NGTDM**	0.91	0.80-0.96
**PET**	1	0.99-1
**Overall radiomics**	0.90	0.62-0.93
**Radiomics + PET**	0.92	0.85-0.96

PET *Positron emission Tomography*, SUV *standard uptake value*, overall radiomics*: sum of all the different matrices*. Radiomics and PET *sum of all the radiomic matrices and PET features*

Supplementary Table 6. Overview of inter-observer variability defined by the intraclass correlation coefficient (ICC), with breakdown between observers statistically assessed using repeated measures ANOVA. Obs = observer. A higher score means a higher correlation: 0.2-0.4 fair agreement, 0.4-0.6 moderate, 0.6-0.8 substantial and >0.8 almost perfect agreement. N=18 patients.

Radiomics feature class	Average ICC	95% CI	Obs 1 vs obs 2 P value	Obs 1 vs obs 3 P value	Obs 2 vs obs 3 P value
FOS	0.93	0.84-0.97	0.81	0.80	0.95
GLCM	0.90	0.79-0.96	0.85	0.94	0.79
GLSZM	0.81	0.60-0.93	0.96	0.94	0.99
NGTDM	0.80	0.23-0.87	0.80	0.76	0.76
PET	0.99	0.98-1	0.92	0.41	1
FOS+GLCM+GLSZM +NGTDM combined	0.86	0.62-0.93	0.85	0.86	0.87
Total radiomics features + PET	0.88	0.69-0.94	0.87	0.77	0.90

PET *Positron emission Tomography*, SUV *standard uptake value*, overall radiomics*: sum of all the different matrices*. Radiomics and PET *sum of all the radiomic matrices and PET features*

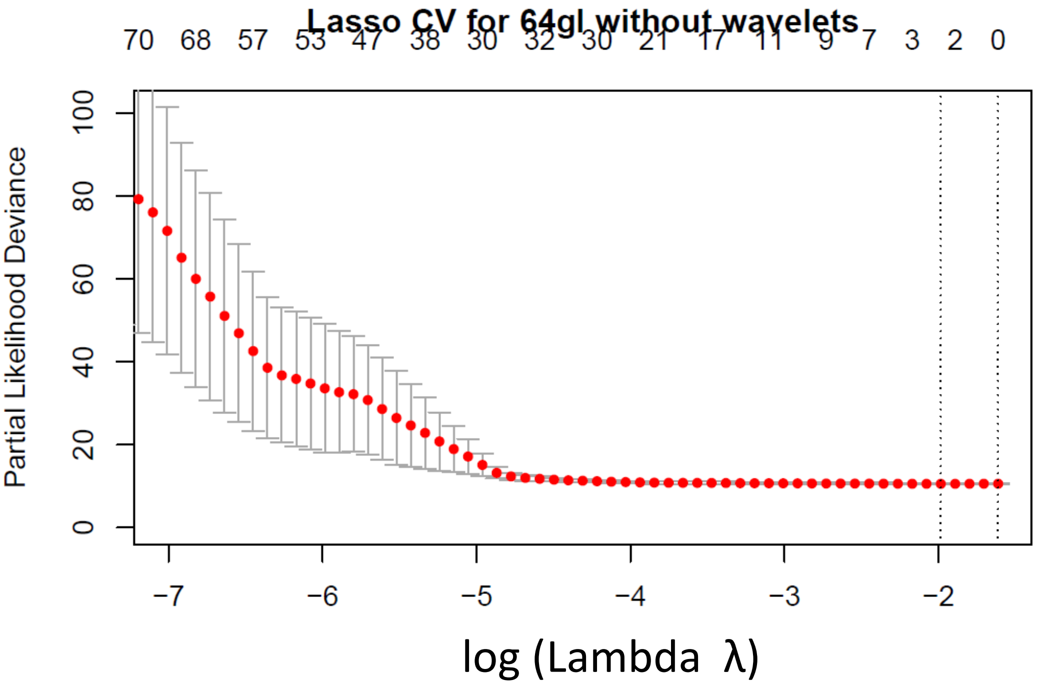

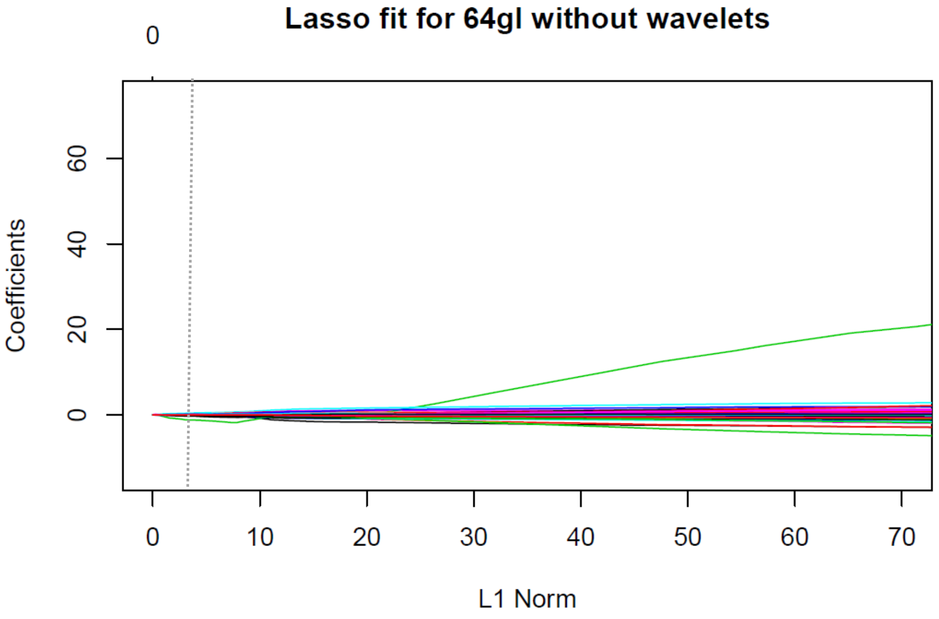

Supplementary Figure 2. Radiomics feature selection using the binary logistic regression model, LASSO. a). The area under the receiver operating curve (AUC) was plotted against the logarithm of the tuning parameter (λ) by determining the minima from cross validation. The optimal values representing x1 SE of the minima are plotted as vertical lines. (B) A LASSO coefficient profile plot showing LASSO coefficients plotted against Normalised values (L1 Norm) is LASSO of the texture features. The vertical line represents the optimal number of non-zero coefficients obtained through cross-validation.

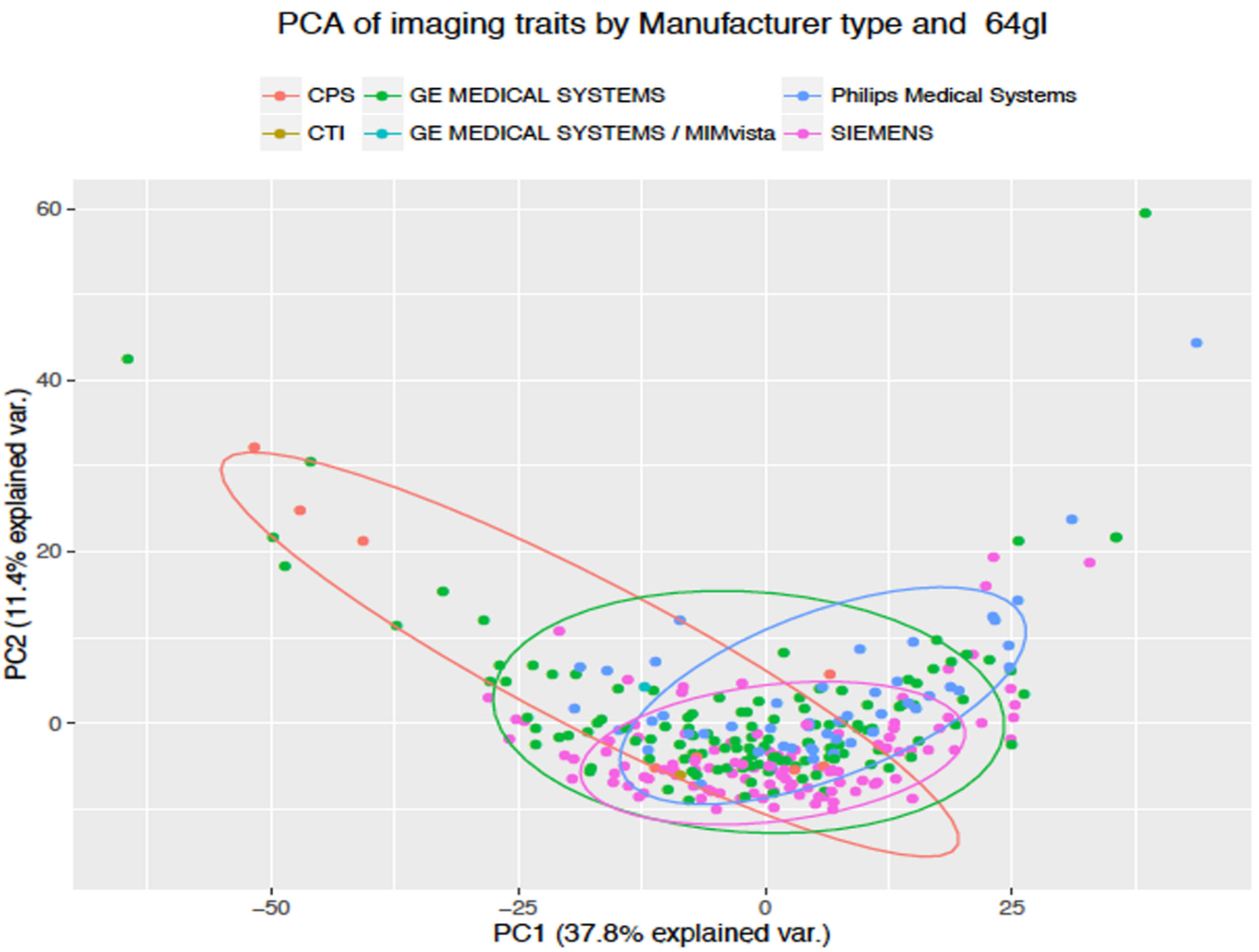

Supplementary Figure 3. Principal component analysis (explained variance) of PET radiomics features (at 64 Gray level) to assess congruence of data from different centers. a) Manufacturer type, including CPS (CTI PET systems), GE Medical Systems, Phillips Medical Systems, CTI, GE Medical systems/MMVista, or Siemens

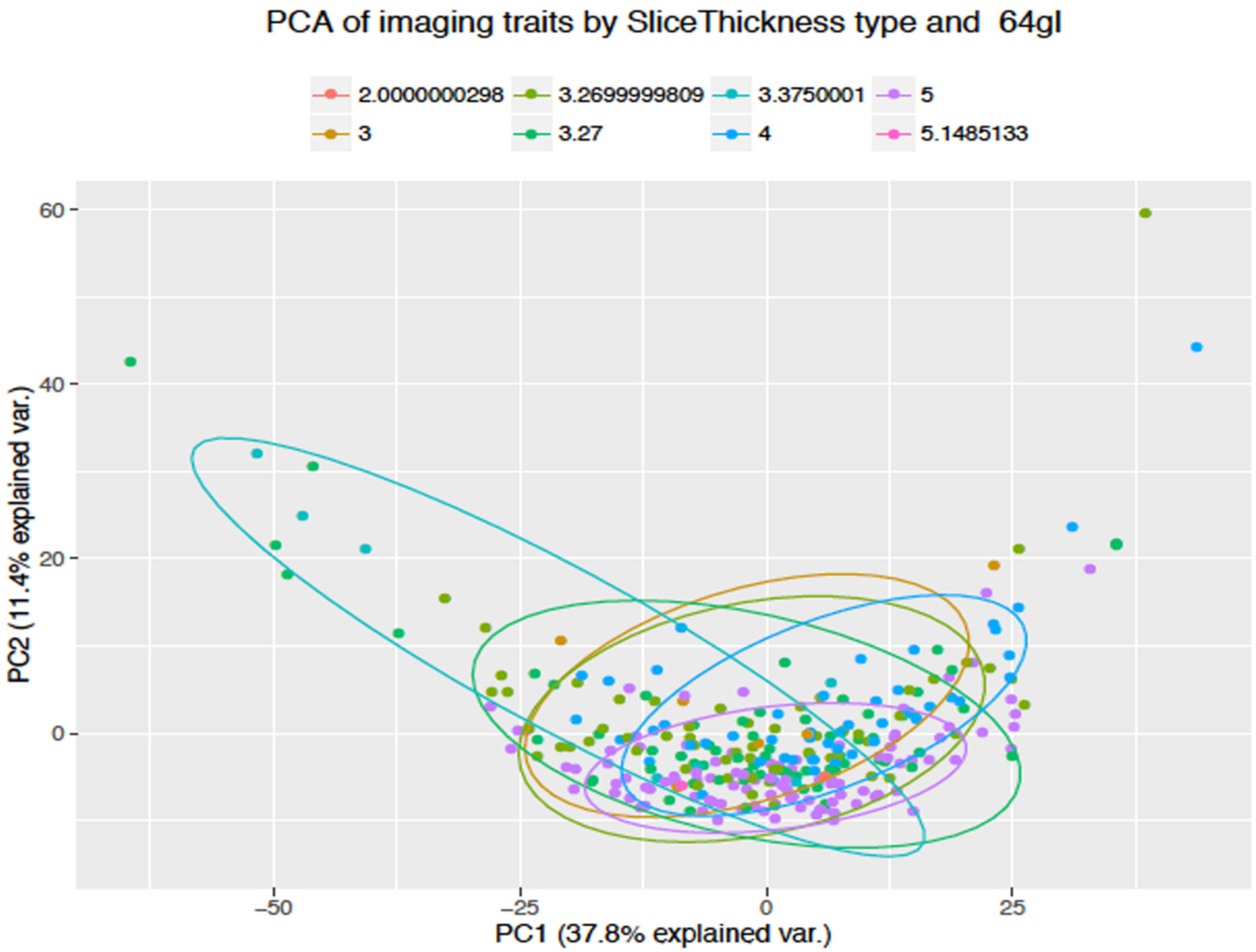

Supplementary Figure 4. Principal component analysis (explained variance) of PET radiomics features (at 64 Gray level) to assess congruence of data from different centers. Slice Thickness: 2, 3, 3.27, 3.3, 3.8, 4, 5, 5.15 mm.

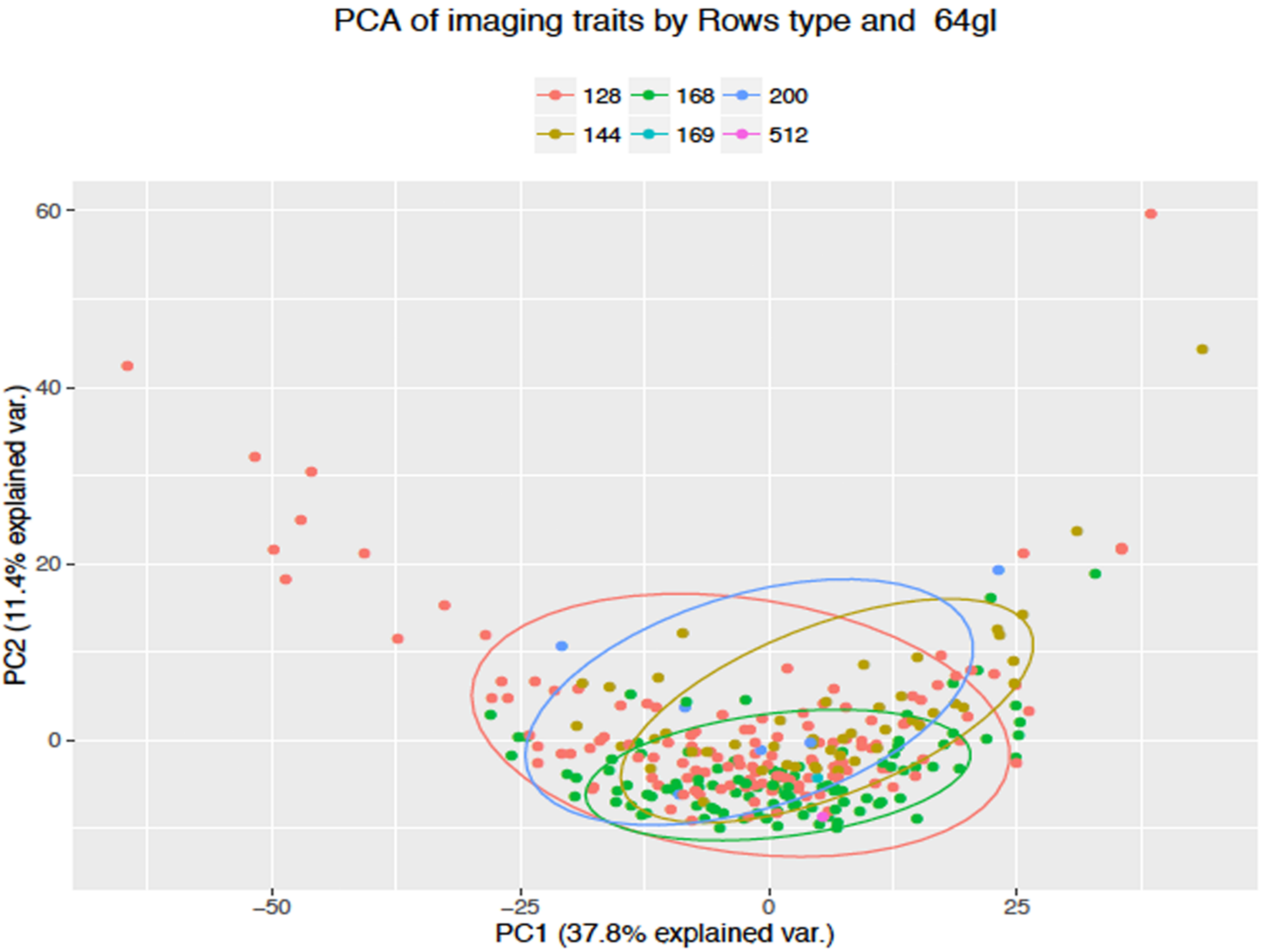

Supplementary Figure 5. Principal component analysis (explained variance) of PET radiomics features (at 64 Gray level) to assess congruence of data from different centers. Number of Rows: 128, 144, 168, 169, 200, and 512. Note the matrix was always symmetrical so the row and column appearances are identical (not shown).

Supplementary Table 7: Correlation between the FVX and various PET model parameters

Parameters	P-value	Spearman correlation coefficient
Model type	0.308*	-
Manufacturer	0.462*	-
Slice Thickness	0.877	0.0135
Rows	0.356	-0.0806
*Calculated from Kruskal-Wallis test		

a)

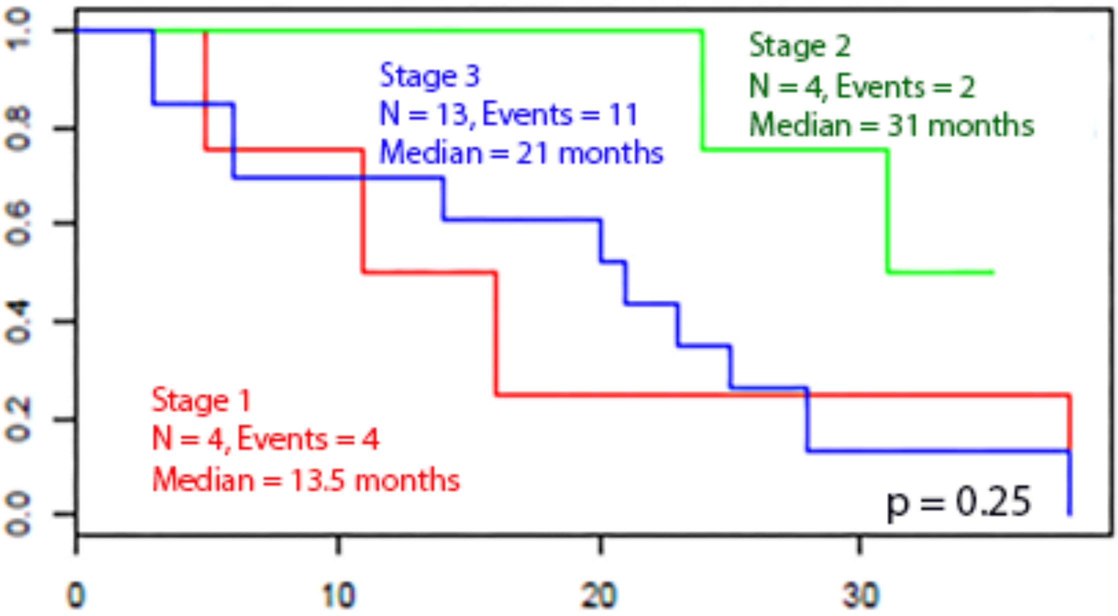

b)

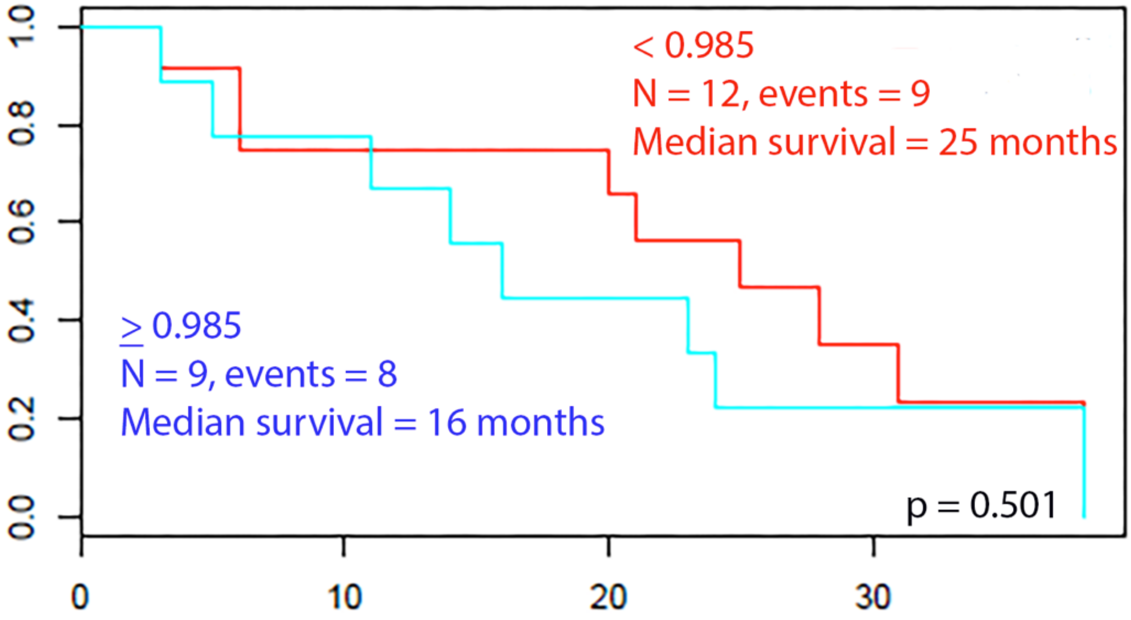

Supplementary Figure 6. a) Overview of the KM plot for stage in TESTI demonstrating poor survival for stage 1 and improved survival for stage 2.
b) KM plot of survival with dichotomizations based on ROC survival. This is not significant.

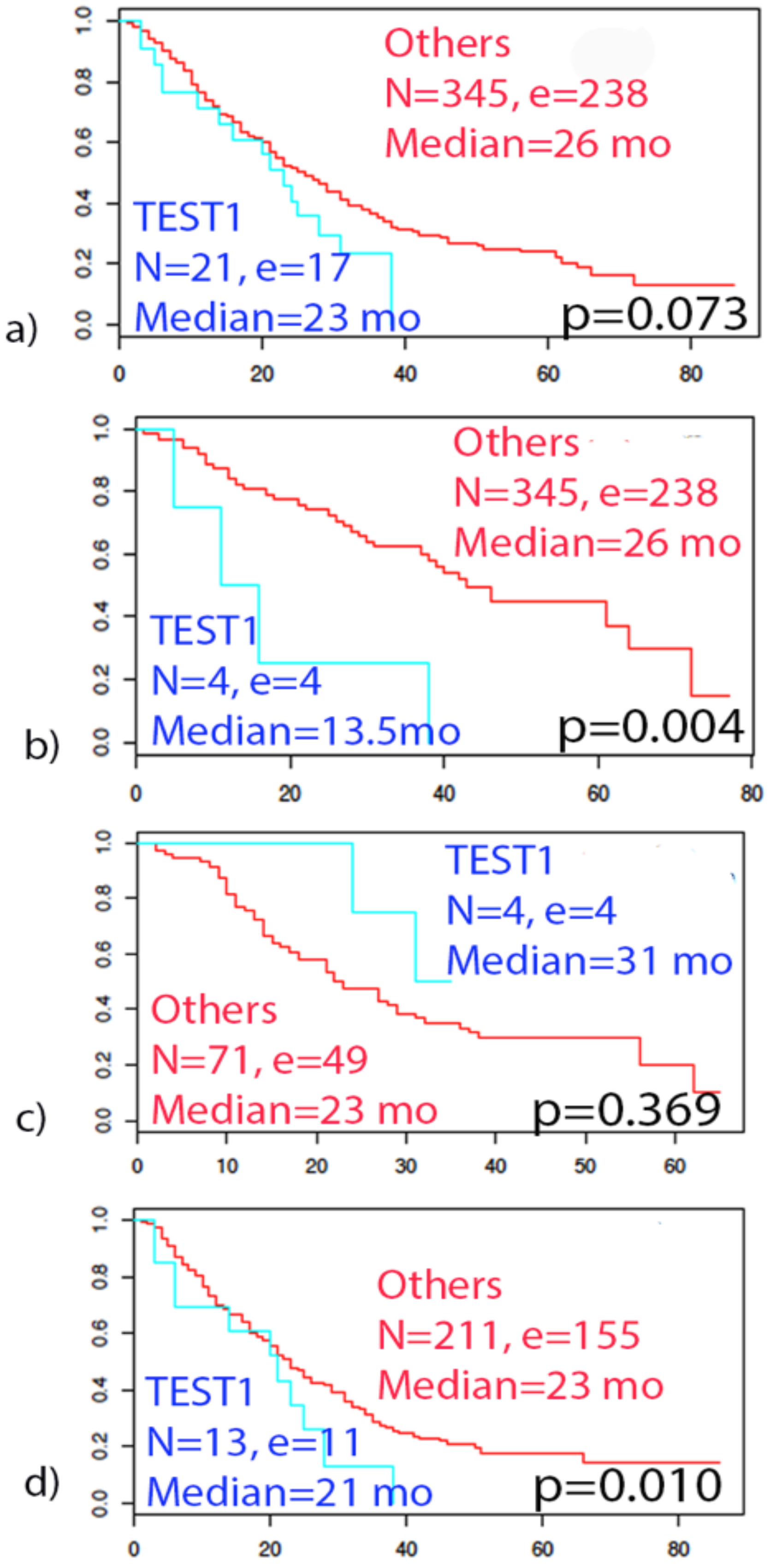

Supplementary Figure 7. KM plots demonstrating survival differences between TESTI and the other subsets combined: a) Overall survival TESTI and the rest, b) Stage 1 differences, c) Stage 2 differences, d) Stage 3 differences.

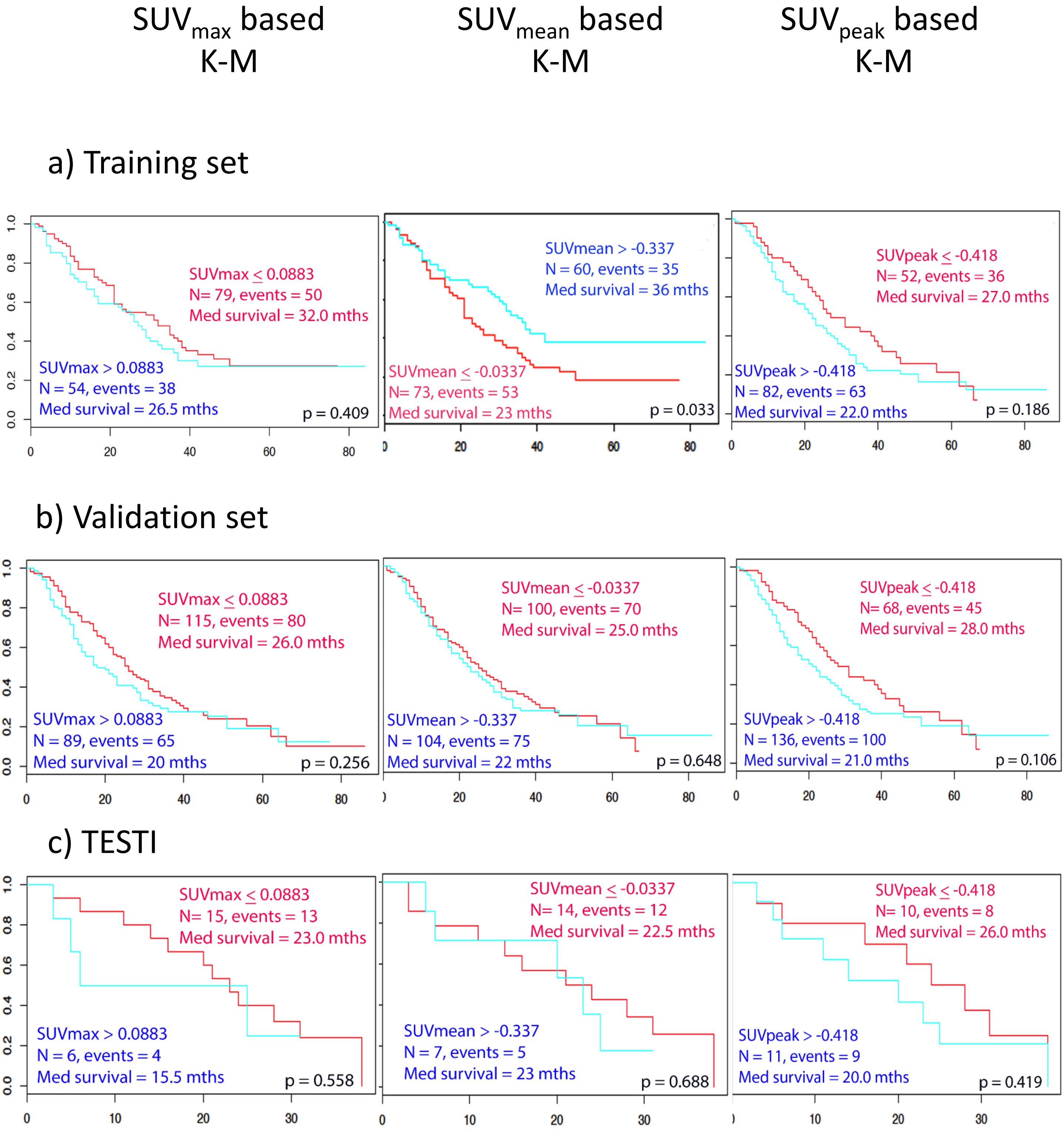

Supplementary Figure 8. Survival analysis based on SUV variables dichotomized using ROC. Kaplan Meier plots of a) training dataset, and b) independent validation set, and c) independent TESTI. Note that the validation dataset has a longer follow-up period.

Supplementary Table 8. Pearson Correlation of the various factors which were positive in the univariate KM plots. The TLG and MTV highly correlated with each other. When either one of them was used in the Cox analysis, they were not significant (results not shown).

	FVX	TLG	MTV	Stage
FVX	1	-0.594	-0.602	-0.283
TLG	-0.594	1	0.844	0.201
MTV	-0.602	0.844	1	0.243
Stage	-0.283	0.201	0.243	1

Supplementary Table 9. Univariate and multivariable analysis of the significant prognosticators with C-index.

		Univariate			Multivariable		
	Variables	HR (95% CI)	P-value	C-index (se)	HR (95% CI)	P-value	C-index (se)
Training (n=133)	FVX	18.9 (3.99-89.3)	0.000213	0.567 (0.034)	19.0 (1.11-323)	0.0419	0.575 (0.034) to 0.595 (0.034)
	Stage	1.55 (1.16-2.08)	0.00335	0.559 (0.031)	1.42 (1.04-1.92)	0.0252	
	MTV	1.25 (1.06-1.48)	0.00989	0.552 (0.034)	0.876 (0.577-1.33)	0.537	
	TLG	1.30 (1.09-1.55)	0.00439	0.546 (0.034)	1.05 (0.697-1.58)	0.814	
Validation (n=204)	FVX	5.30 (1.69-16.6)	0.00429	0.588 (0.027)	9.62 (1.35-68.7)	0.024	0.562 (0.027) to 0.579 (0.027)
	Stage	1.22 (0.975-1.52)	0.0834	0.55 (0.023)	1.12 (0.881-1.43)	0.352	
	MTV	1.07 (0.939-1.23)	0.304	0.593 (0.027)	0.752 (0.505-1.12)	0.160	
	TLG	1.12 (0.983-1.27)	0.0915	0.602 (0.027)	1.16 (0.822-1.65)	0.394	
Test1 (n=21)	FVX	0.187 (0.00306-11.5)	0.425	0.547 (0.088)	1595 (0.00912-2.79e8)	0.231	0.541 (0.088) to 0.558 (0.088)
	Stage	0.998 (0.536-1.86)	0.995	0.506 (0.08)	1.02 (0.515-2.03)	0.951	
	MTV	0.535 (0.210-1.37)	0.192	0.552 (0.088)	0.165 (0.00471-5.81)	0.322	
	TLG	0.434 (0.110-1.71)	0.232	0.547 (0.088)	0.586 (0.0107-32.1)	0.794	
